# The Microbiology of Postoperative Spine Surgical Site Infections: An Institutional Experience of 170 cases

**DOI:** 10.1016/j.jcot.2026.103553

**Published:** 2026-07-11

**Authors:** Audrey Zhao, Westin Mace, Jonathan Tran, Saahil Sandhar, Hania Shahzad, Wyatt Vander Voort, Safdar Khan, Hai Le

**Affiliations:** Department of Orthopaedic Surgery, University of California, Davis, Sacramento, CA, 95817, USA

**Keywords:** SSI, Microbiology, Postoperative spine infection, Intraoperative culture, Chronic antibiotic therapy

## Abstract

**Background:**

Postoperative spine infections are surgical site infections that develop following spine surgery and often require operative management to reduce infection burden and provide source control. Intraoperative cultures are essential for identifying pathogens and guiding antimicrobial therapy. We characterized the microbiology and antimicrobial treatment of patients with postoperative spine infections at our institution.

**Methods:**

We performed a retrospective review of patients with postoperative spine surgical site infections (SSI) requiring surgical intervention between 2015 and 2025. Intraoperative culture data and postoperative antibiotic regimens were recorded. Associations between chronic antibiotic suppression and presence of an extra-spinal infection source, microbiologic concordance between extra-spinal and intraoperative cultures, and polymicrobial infection were evaluated.

**Results:**

Between 2015 and 2025, 170 of 4921 patients (3.5%) underwent surgical management for postoperative spine SSI. Mean age was 58.7 years (SD 18.3), with 93 females (55%). Of these, 112 were elective (65.9%) and 113 were primary surgeries (66.5%). Patients had a mean of 2 reoperations (SD 1.9). 111 patients (65.3%) had positive intraoperative cultures. Among them, 62 infections were monomicrobial (55.9%), with 14 MRSA and 18 MSSA. Of the 170 cases, 38 (22.4%) had an extra-spinal infection source, with blood (19, 50%) and urine (22, 57.9%) being most common. Of the 111 patients with positive cultures, 29 (26.1%), had an extra-spinal infection source, and nearly half had overlapping microbiology (13, 44.8%). 35 patients (20.6%) required lifelong oral antibiotics. Chronic antibiotic therapy was significantly associated with presence of an extra-spinal infection source, but not organism concordance or polymicrobial infection.

**Conclusion:**

Among 170 patients with postoperative spine SSI, two-thirds had positive intraoperative cultures, with MRSA and MSSA being most common. High rates of concordance were observed between extra-spinal infection sources and intraoperative cultures. Still, one-fifth of patients required lifelong oral antibiotics for infection control.

## 1 Introduction

Surgical site infections (SSIs) that develop after spine surgery are the third most common complication following spine surgery.^1^ Pooled incidence of postoperative spine infection by meta-analysis is reported to be 3%, with a rate ranging from 0.2% to 16.1% in individual studies, depending on the patient population.^2^ Despite advances in infection management, SSIs remain challenging to diagnose and treat and have been associated with considerable long-term consequences, including mortality rates as high as 15% at five years postoperatively.^3^

The spine is particularly susceptible to infection due to its rich vascular network, permitting hematogenous seeding, and its deep anatomic location, which may limit early detection and effective source control.^4^ Patient-related factors that impair wound healing–including uncontrolled diabetes, obesity, and prolonged corticosteroid use–have been consistently associated with infection risk.^5^^,^^6^ Surgical factors, including longer operative duration and posterior approaches, are associated with greater tissue disruption and prolonged wound exposure. Instrumentation, a cornerstone of many spinal procedures, further increases susceptibility by providing an avascular surface that facilitates bacterial adherence and biofilm formation.^7^ The consequences of SSI can be severe, resulting in prolonged hospitalization, implant failure, or loss of spinal stability, and delayed return to function, all of which increase healthcare utilization and overall system burden.^8^^,^^9^

Early symptoms of SSI may be difficult to distinguish from routine postoperative pain, and management is often complex in spine surgery patients who frequently have significant comorbidities. Initial treatment typically involves intravenous (IV) antibiotic therapy tailored to organism identification and susceptibility testing.^10^ In hemodynamically stable patients, antibiotic treatment is typically deferred until cultures are obtained to maximize microbiological yield. However, broad-spectrum empiric antibiotic treatment is initiated immediately when there is suspected neurologic compromise or systemic infection.7 Although nonoperative treatment can be effective, it is not always sufficient for patients with extensive infection or poor baseline health status. Surgical management is therefore necessary to reduce the infectious burden and provide source control.

Existing literature identifies *Staphylococcus aureus*, *Enterobacterales*, and coagulase-negative staphylococci as the predominant pathogens in postoperative spinal SSI, but contemporary data describing microbiologic trends and management paradigms remain limited.^11^ Given the evolving epidemiology of SSI and the increasing volume of spine procedures performed annually, a detailed understanding of organism distribution in postoperative infection is critical to inform management strategies. The objective of this study was to characterize the microbiology of 170 patients with postoperative spine infection at our institution and to describe patterns of antimicrobial treatment.

## 2 Methods

Following IRB approval (IRB-ID 2139637-1), we reviewed our institution's database of 4921 adult patients who underwent spine surgery between 2015 and 2025. Of these, 170 (3.45%) patients developed a postoperative SSI requiring surgical management. Patients who were treated nonoperatively and those with primary spine infections or revision surgery for other causes, such as seroma formation, were excluded.

The data from this study was collected from a database of orthopedic spine surgeons at an academic medical center in an urban setting. The cohort included both instrumented and non-instrumented spinal procedures, including decompression-only procedures such as laminectomy and discectomy.

A retrospective chart review was conducted, and demographic variables were collected, including age, sex, race, and ethnicity. Surgical data abstracted included operative urgency (elective vs non-elective) and whether the index procedure was a primary or revision operation. Non-primary procedures included revision operations, staged procedures, and unplanned returns to the operating room, as documented in the medical record. Diagnosis of SSI was determined based on a chart review of clinical findings and documentation of surgical intervention for infection treatment.

### 2.1 Microbiological methods

At our institution, intraoperative specimens are routinely obtained in patients undergoing surgical management of postoperative spine SSI, with the number of specimens varying from one to three by case. Perioperative cefazolin is typically administered, and vancomycin powder is applied before closure. Our institution utilizes a diluted betadine solution for final irrigation of the surgical site. Vancomycin and cefazolin are initiated for broad spectrum coverage when infection is suspected and not routinely withheld prior to culture collection. Cultures are processed according to institutional protocols, typically with incubation for up to seven days; anaerobic and fungal cultures are obtained based on clinical suspicion. Hardware sonication is not routinely performed. Low-virulence organisms commonly considered skin flora, including Cutibacterium acnes and coagulase-negative staphylococci, were not adjudicated using predefined criteria. Interpretation as pathogens versus contaminants was based on clinical documentation and treating team assessment as recorded in the medical record.

Postoperative antibiotic treatment plans provided by the infectious disease team were obtained, including the need for chronic suppressive antibiotic therapy. Extra-spinal sources of infection, such as blood and urine, were noted. For outcome variables, the number of reoperations, readmissions, and emergency department (ED) visits, and the total length of stay (LOS) across spine-related admissions were recorded. Readmission included only surgical complications such as pseudarthrosis and infection. Medical complications leading to readmission were excluded. Procedures such as hardware removal, wound vacuum-assisted closure (wound VAC), and final wound closure by plastics were also documented.

### 2.2 Statistical analysis

Descriptive statistics were computed for all baseline patient variables, surgical characteristics, and outcomes. Mean and standard deviation or median with interquartile ranges (IQR) were used for continuous variables. Chi-square analysis and Fisher's Exact Test were used to evaluate if presence of an extra-spinal infection source, a concordant organism identified in an extra-spinal source, or polymicrobial infection were associated with the need for chronic antibiotic suppression.

## 3 Results

### 3.1 Demographics

During the study period of 2015-2025, 170 out of 4921 patients (3.5%) developed postoperative spine infections requiring surgical intervention. The mean age was 58.7 years (SD 18.3), and the cohort included 93 females (55%)(Table 1).

**Table 1:** Descriptive Demographic and Surgical Cohort Data

Variable	n (%) or Mean ± SD
**Demographics**	
Age	58.7 ± 18.3
Sex	
Female	93 (55)
Male	77 (45)
**Surgical Characteristics**	
Primary Surgery	114 (67)
Elective Surgery	112 (65.9)
Instrumented	150 (87.7)
**Postoperative Management**	
Wound VAC used	53 (31)
Plastic Surgery Closure	25 (14.6)
Hardware Removal	48 (32)
Reoperations	2 ± 1.9
Readmissions	2.1 ± 2.6
Total Length of Stay (all admissions)(days)	18 ± 35.5

Abbreviations: VAC; vacuum assisted closure.

### 3.2 Surgical characteristics and postoperative management

Overall, 112 of the 170 patients underwent elective surgeries (65.9%), and 113 (66.5%) were primary procedures. A total of 88.2% of the patients with SSI had instrumentation and all implants were present at the time of SSI diagnosis (150/170). Of these, 48 patients (32%) eventually had hardware removal. Patients underwent an average of 2 subsequent reoperations following the initial procedure. The average number of readmissions was 2.1, with an average LOS of 18 days across all admissions. Staged surgery with wound vacuum-assisted closure (VAC) was performed in 53 patients (31%), and plastics completed the final wound closure in 25 patients (14.7%)(Table 1).

### 3.3 Microbiology

**Fig. 1:**
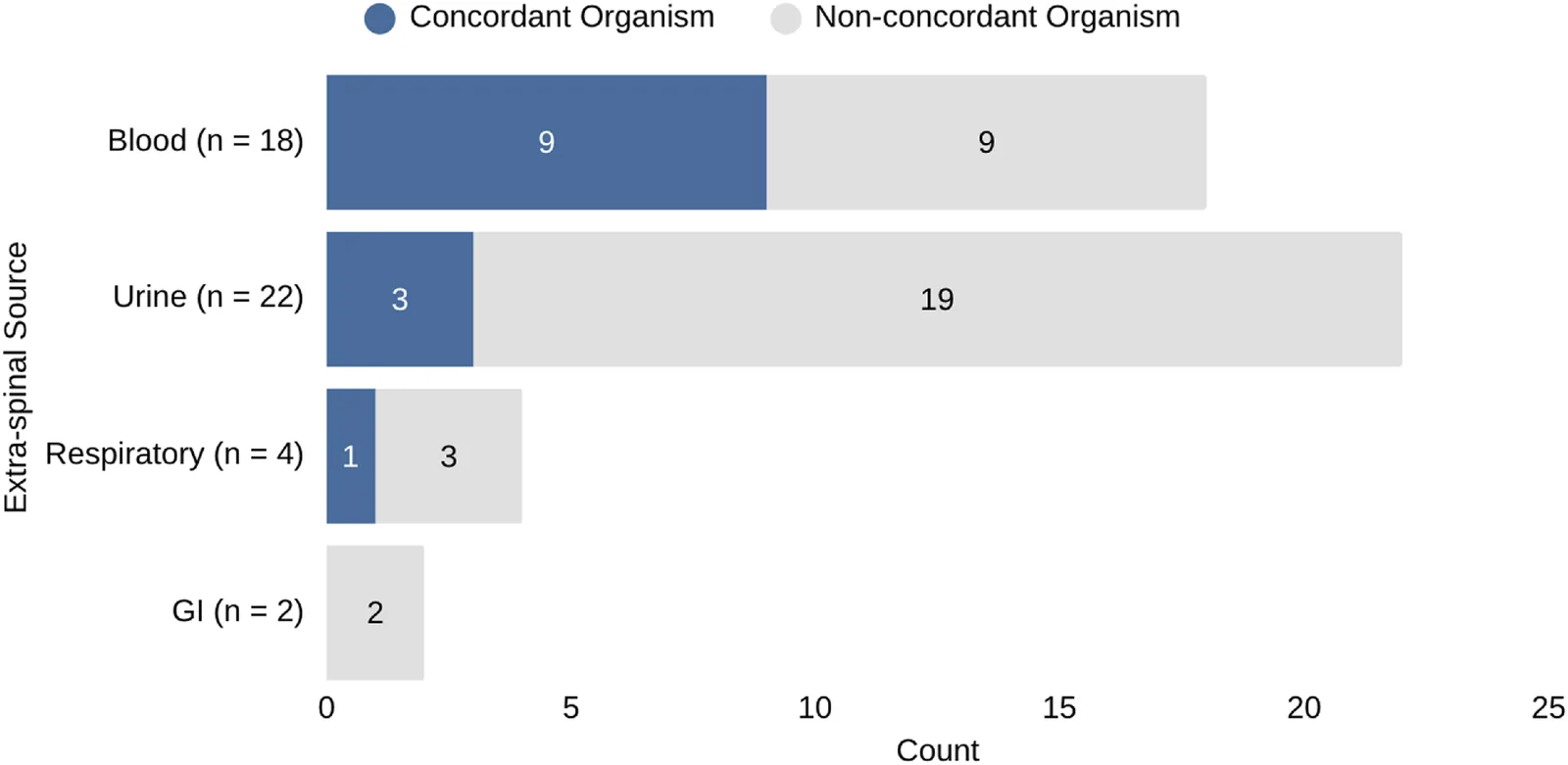
Microbiologic Concordance with Intraoperative Cultures in Extra-spinal Infection Sources Bars represent the number of cases in which organisms isolated from extra-spinal infection sources were concordant or non-concordant with organisms identified in intraoperative cultures. Blood cultures demonstrated the highest rate of concordance with intraoperative cultures. Note: 19 patients had blood as an extra-spinal infection source, but concordance was evaluated for only 18 patients, as 1 patient had a negative intraoperative culture. GI = gastrointestinal.

**Fig. 2:**
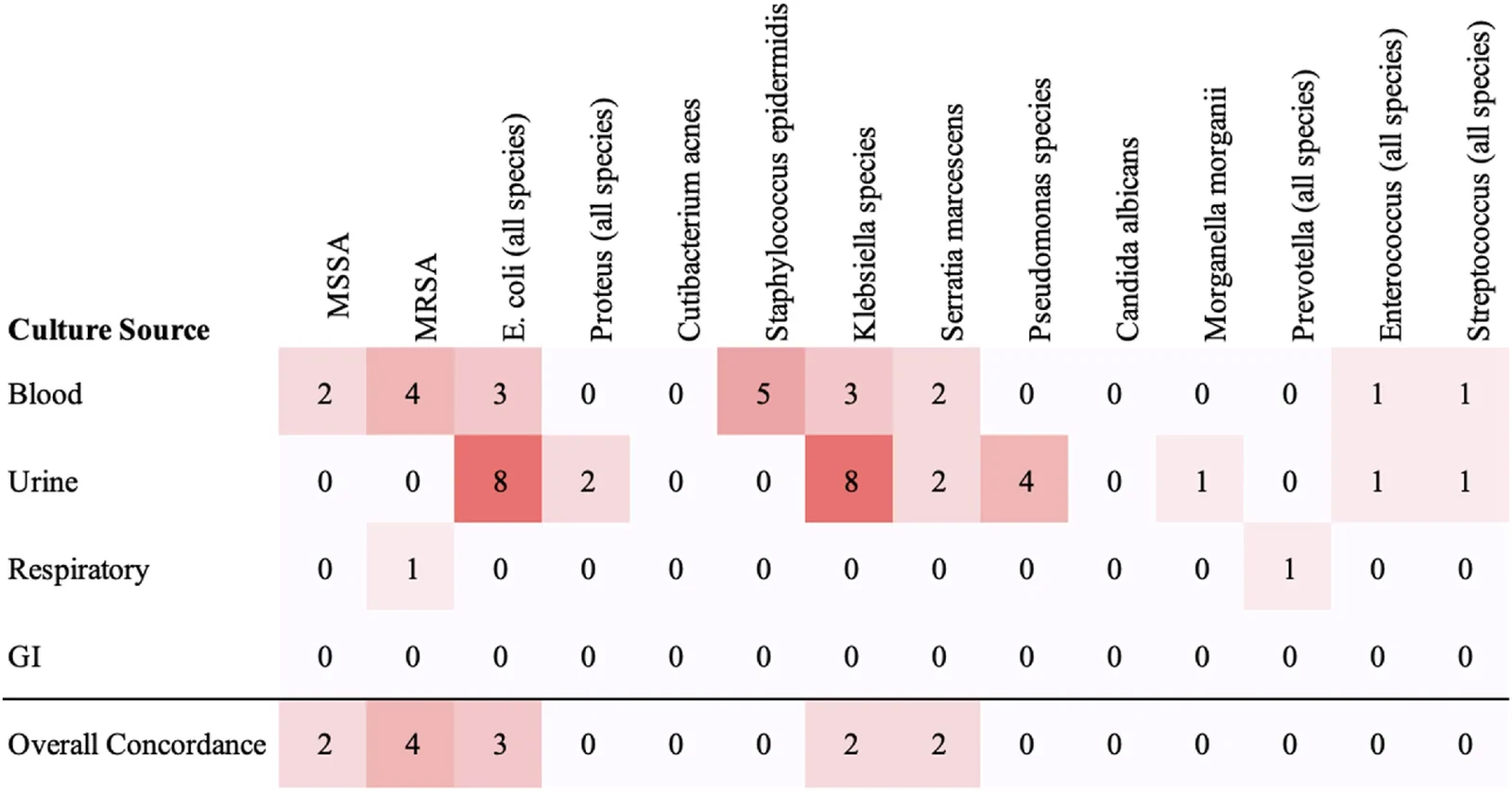
Distribution of Organisms across Extra-spinal Infection Sources Cells represent the frequency with which each organism was isolated from blood, urine, respiratory, or gastrointestinal sources. Darker shading indicates higher organism frequency. The bottom row highlights organisms demonstrating the highest concordance with the organisms identified in intraoperative cultures. MSSA, Methicillin-Susceptible *Staphylococcus aureus*; MRSA, Methicillin-Resistant *Staphylococcus aureus*; *E. coli*, *Escherichia coli*; GI, gastrointestinal.

Positive intraoperative cultures were found in 111 patients (65.3%). Of these, 62 (55.9%) were monomicrobial, with 14 MRSA and 18 MSSA infections (Table 2). Among the monomicrobial infections, Gram-positive organisms predominated (55.6%), followed by Gram-negative bacteria (27.0%), anaerobes (7.9%), and other rare organisms (9.5%). In the overall cohort of 170, an extra-spinal source of infection was identified in 38 patients (22.4%), with blood (19 patients) and urine (22 patients) being the most common sources (Fig. 1). There were 29 patients (26.1%) with a positive intraoperative culture who had an extra-spinal infection source; 13 (43.3%) of these had overlapping microbiology with the organism identified on intraoperative culture (Fig. 2).

**Table 2:** Most Common Organisms Isolated from Intraoperative Cultures

Organism	Total Cultures (n)	Monomicrobial Cultures (n)
**Gram-positive**		
***Staphylococcus aureus* (all species)**	58	33
MSSA	31	18
MRSA	25	14
*S. aureus* (unspecified)	2	1
Cutibacterium acnes	9	4
*Staphylococcus epidermidis*	8	3
**Enterococcus (all species)**	8	1
Enterococcus (VRE)	5	1
**Streptococcus (all species)**	6	1
Group B Streptococcus	2	1
*Streptococcus anginosus*	2	0
Streptococcus viridans	1	0
Group G Streptococcus	1	0
**Gram-Negative**		
***E. coli* (all species)**	13	6
*Escherichia coli* (non-ESBL/CRE)	9	3
*Escherichia coli* (ESBL)	2	2
*Escherichia coli* (CRE)	1	1
Klebsiella species	13	3
**Proteus (all species)**	11	4
*Proteus mirabilis*	4	3
Proteus (unspecified)	7	1
Pseudomonas species	9	2
*Serratia marcescens*	3	3
**Prevotella (all species)**	3	1
Prevotella biva	1	1
*Morganella morganii*	1	1
**Fungal**		
*Candida albicans*	5	1

Abbreviations: MSSA, Methicillin-Susceptible *Staphylococcus aureus*; MRSA, Methicillin-Resistant.

*Staphylococcus aureus*; *S. aureus*, *Staphylococcus aureus*; *E. coli*, *Escherichia coli*; ESBL.

Extended-speectrum beta-lactamases; CRE, Carbapenem-resistant; VRE, Vancomycin-resistant Enterococci.

### 3.4 Antibiotic therapy

The mean and median antibiotic treatment durations were 72 (SD 143) and 42 days (IQR 10, 84), respectively, and patients required a mean of 2.5 antibiotics during treatment. Of the 170 patients, 149 (87.6%) were treated with intravenous (IV) and 75 (44.1%) received oral antibiotics. Nearly two-thirds of patients received two or more antibiotics, and 20.6% required chronic suppressive antibiotic therapy. Of these, doxycycline (40%) and sulfamethoxazole/trimethoprim (17%) were the most commonly prescribed (Table 3). Among patients who did not have chronic suppressive therapy, the most commonly utilized antibiotics were vancomycin (n = 74), cefazolin (n = 53), rifampin (n = 22), levofloxacin (n = 15), and trimethoprim-sulfamethoxazole (n = 15).

**Table 3:** Chronic Suppressive Antibiotic Use

Antibiotic Name	Total
Doxycycline	15
TMP/SMX	6
Minocycline	3
Levofloxacin (incl. Levaquin)	3
Augmentin	3
Cephalexin	2
Rifampin	2
Dicloxacillin	1
Mycobutin	1
Moxifloxacin	1
Linezolid	1
Omadacycline	1
Ritonavir	1

Abbreviations: TMP/SMX, Trimethoprim/Sulfamethoxazole.

Chronic suppressive antibiotic therapy was significantly associated with the presence of an extra-spinal source of infection (OR = 2.6, 95% CI 1.05-6.25, p = 0.02). No significant association was observed with polymicrobial intraoperative culture (OR = 1.04, 95% CI 0.4-2.64, p = 0.99) or concordance between extra-spinal and intraoperative cultures (OR = 0.74, 95% CI 0.27-5.16, p = 0.74)(Table 4).

**Table 4:** Factors Associated with Chronic Suppressive Antibiotic Use

Concordant Organism Present	Chronic Antibiotic Use	None	Total	OR [95% CI]	p-value
Yes	4	9	13	1.29 [0.27-5.16]	0.74
No^a^	25	73	98		
Total	29	82	111		
**Secondary Infection Present**					
Yes	13	25	38	2.6 [1.05-6.25]	**0.02**
No^a^	22	110	132		
Total	35	135	170		
**Polymicrobial Infections**	13	36	49	1.04 [0.4-2.64]	0.99
**Monomicrobial Infections**	16	46	62		
Total	29	82	111		

a Used as reference group.

## 4 Discussion

This retrospective study characterizes the microbiology and clinical presentations of 170 postoperative spine infections requiring surgical intervention. Postoperative spine SSI resulted in a substantial healthcare burden in our cohort, frequently requiring a complex course of reoperations and hospital readmissions. MRSA and MSSA remained the most prevalent pathogens in our data, which is consistent with prior literature, and one in five patients required chronic suppressive antibiotic therapy. An extra-spinal infection source was identified in 22.4% of patients, and nearly half of those demonstrated overlapping microbiology with intraoperative cultures, indicating a potential hematogenous contribution in a meaningful subset. Overall, these findings raise the possibility that factors beyond the primary infecting organism may influence infection complexity and treatment burden.^8^^,^^12^^,^^13^

**Fig. 3:**
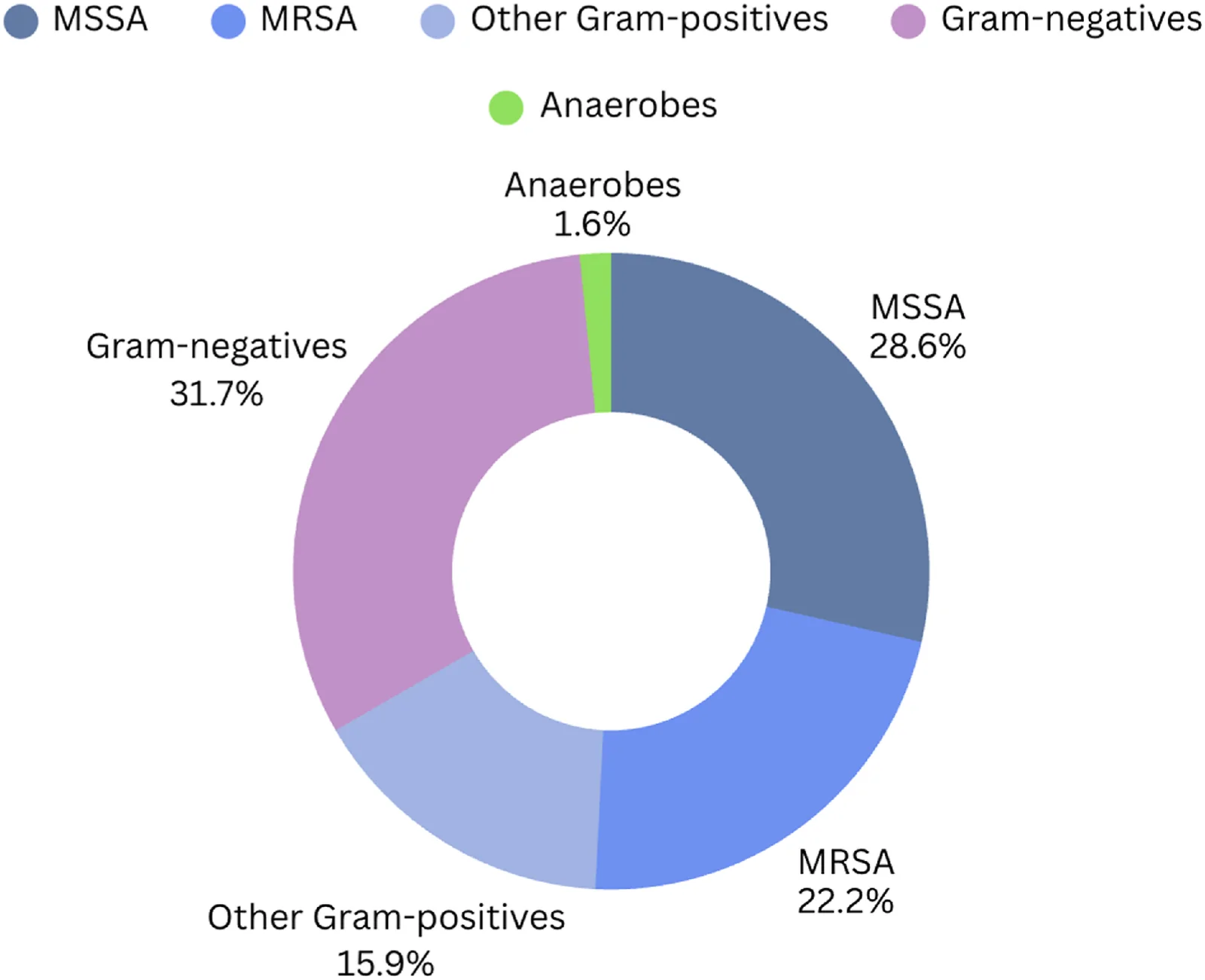
Monomicrobial Culture Distribution The frequency of microorganisms identified in monomicrobial cultures is displayed. MRSA and MSSA comprised half of all monomicrobial cultures. Gram-negatives were well-represented in the culture data. MSSA, Methicillin-Susceptible *Staphylococcus aureus*; MRSA, Methicillin-Resistant *Staphylococcus aureus*.

Our intraoperative culture data demonstrated that 55.9% of infections were monomicrobial. Although 26 unique species were identified, *Staphylococcus aureus* predominated, accounting for 50.8% of monomicrobial cultures (18 MSSA, 14 MRSA isolates). *S. aureus* remains the most common pathogen, likely secondary to its prevalence in normal skin flora, high rate of bacteremia, and ability to form biofilm on instrumentation.^7^ The literature also reports an increasing prevalence of gram-negatives, identifying enterobacteriaceae in up to 55.4% of cultures.^6^^,^^12^ Gram-negatives were well-represented in our findings, comprising over a quarter of monomicrobial infections. Among Gram-negatives, *E. coli* and *Proteus* species were the most commonly identified (Fig. 3) .

**Fig. 4:**
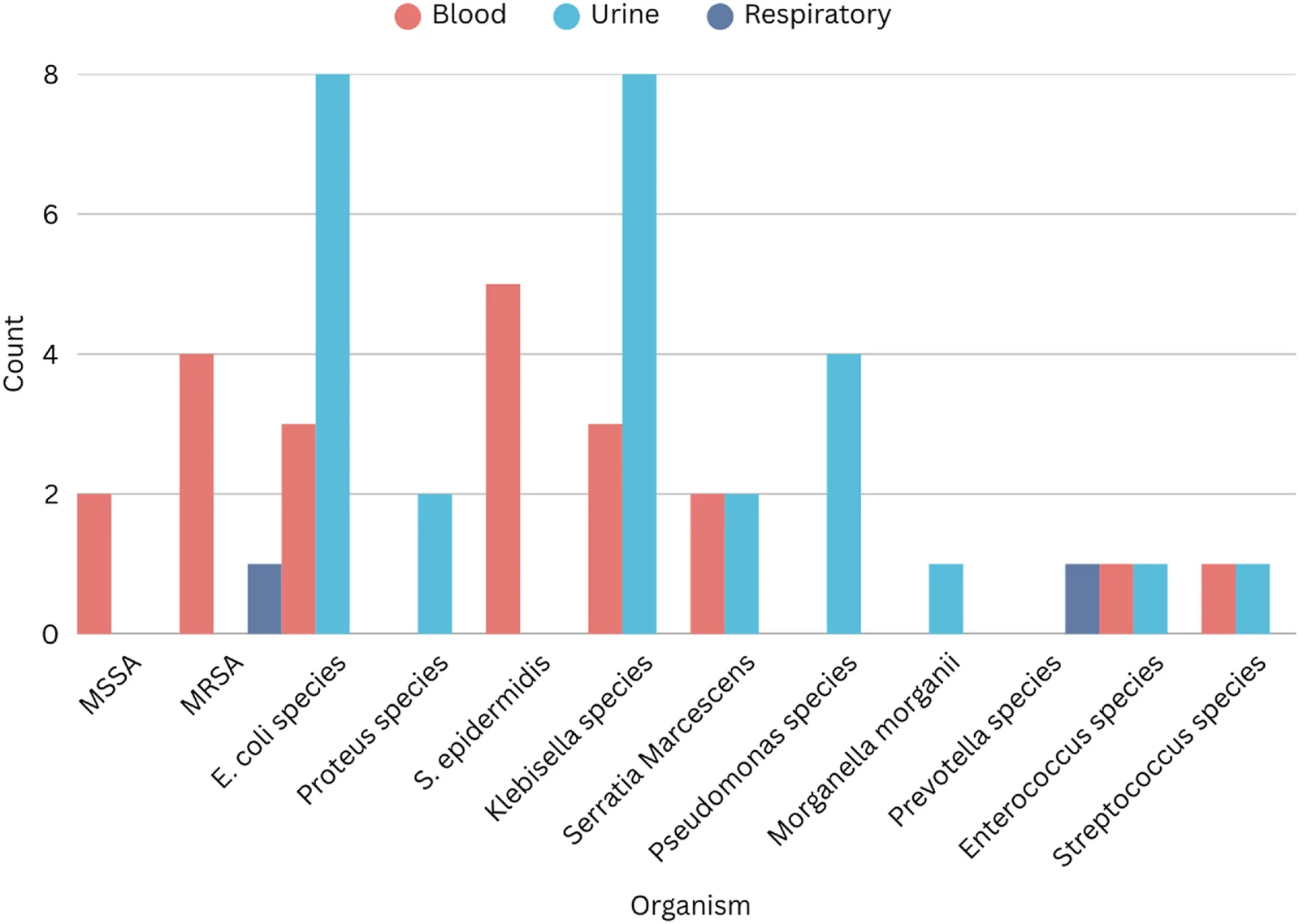
Extra-spinal Infection Source Organism Breakdown The frequency of microorganisms identified in extra-spinal infection sources is displayed. *E. coli* and Klebsiella, mostly identified in urine cultures, were the most common organisms. MSSA, Methicillin-Susceptible *Staphylococcus aureus*; MRSA, Methicillin-Resistant *Staphylococcus aureus*; *E. coli*, *Escherichia coli*; *S. epidermidis*, *Staphylococcus epidermidis*.

Evaluation of extra-spinal infection sources permitted concordance analysis with intraoperative cultures, helping distinguish clinically relevant pathogens from organisms identified across multiple culture sites (Fig. 4). At least one identified organism matched the intraoperative culture in 27% of patients with positive urine cultures. While causality cannot be established, the urinary and gastrointestinal tracts may represent potential sources of infection given their frequent colonization by gram-negative bacteria.^14^ The highly vascularized vertebral structures are also susceptible to hematogenous spread of infection. As expected, the pathogens identified in blood cultures had the highest concordance with intraoperative species (50%). These findings support the perioperative evaluation of extra-spinal sources of infection, such as indwelling catheters and poorly controlled diabetes. Routine assessment for bacteremia or urinary tract infection, particularly in delayed or recurrent cases, may assist in identifying potential hematogenous contributors and guide targeted antimicrobial therapy. The observed presence of gram-negative and anaerobic organisms in our cohort suggests that these pathogens should be considered in empiric antimicrobial selection; however, specific treatment recommendations cannot be made based on these data alone (Fig. 3).

Chronic suppressive antibiotic use is not well-studied in spine surgery but has been reported to range from 5% to 14% in total joint arthroplasty patients for treatment of periprosthetic joint infection.^15^ One-fifth of the patients in our cohort required chronic suppressive antimicrobial therapy, most commonly with doxycycline and trimethoprim-sulfamethoxazole. Consistent with the standard six-week course typically recommended for postoperative spine infection, the median treatment duration was 42 days.^16^ The high prevalence of retained instrumentation provides a surface for biofilm formation, impairing host immune clearance and limiting antibiotic penetration, and frequently necessitating prolonged antibiotic therapy.^17^ The proportion of patients requiring chronic suppressive antibiotics likely contributed to the higher mean treatment duration. This finding may reflect the inherently complex infections present in our cohort, including recurrent infection or antimicrobial-resistant organisms, given that only patients requiring operative management were included in this study.

While the microbiological profile of our cohort was consistent with prior literature, we performed exploratory analyses to evaluate factors associated with chronic antibiotic therapy use observed in our study. The presence of an extra-spinal infection source was associated with chronic antimicrobial therapy, whereas polymicrobial intraoperative cultures and organism concordance were not. These findings differ from prior reports suggesting that polymicrobial SSIs are more difficult to treat due to recurrence and increased antibiotic resistance.^12^^,^^18^ However, these findings should be interpreted cautiously and viewed as associative rather than causal. The observed association with extra-spinal infection sources may reflect greater overall clinical complexity or unmeasured confounding rather than a direct relationship. In addition, clinically relevant factors such as the number of debridements and infection chronicity were not consistently available and may influence the need for chronic suppressive therapy. Further investigation is needed to better identify factors associated with chronic antibiotic therapy, which may help identify at-risk patients earlier in their course, guide surgical decision-making for source control, and clarify the long-term burden of postoperative spine SSIs on patients and healthcare systems.

### 4.1 Limitations

This study has several limitations. First, the data were derived from a single tertiary care institution, which may limit generalizability despite overall consistency with prior reports. Future investigation should include data from multiple centers to evaluate these findings across broader patient populations. This was a retrospective study, which may limit information regarding specific indications for reoperation, preoperative diagnoses, staged procedure timing, and other details affecting management strategy. Detailed characterization of mortality and readmissions, including timing and cause, was not consistently available, limiting assessment of the true systemic and economic burden of infection. Additionally, at the time of data collection, no standardized definition of spine infection existed. Diagnosis was determined based on a combination of documented clinical findings as well as intraoperative data, which may introduce variability in clinical judgment and treatment thresholds. Microbiologic interpretation was not standardized, and low-virulence organisms (e.g., *Cutibacterium acnes* and coagulase-negative staphylococci) were not systematically adjudicated as contaminants, potentially overestimating infection rates. Although efforts were made to include outcomes such as reoperation, readmission, and antibiotic use directly attributable to spine infection, these outcomes may be over- or underestimated given the multifactorial nature of postoperative care and patient comorbidities. Finally, our study did not evaluate outcomes stratified by infection timing, intraoperative culture results, or specific organisms. In addition, key variables required to compare culture-positive and culture-negative infections, including preoperative antibiotic exposure and infection chronicity, were not consistently available, precluding meaningful subgroup analysis. Other granular comorbidity data, such as control of diabetes and degree of immunosuppression, were also not available for analysis in this retrospective dataset. Future efforts should build upon our findings using prospective designs and these granular clinical data to better define strategies for improving postoperative spine infection management.

### 4.2 Conclusion

Our findings underscore the importance of ongoing institutional microbiologic surveillance and careful evaluation of potential extra-spinal infection sources to inform treatment strategies. As spine surgery volumes continue to increase, the burden of postoperative SSI is expected to rise accordingly. Postoperative spine infections represent a significant clinical and economic burden, characterized by high rates of reoperation and prolonged hospitalizations. Our findings reveal that over 20% of patients require lifelong suppressive antibiotic therapy, highlighting a level of long-term morbidity that necessitates early multidisciplinary intervention and aggressive source control. Unlike other areas of orthopedics, standardized diagnostic and management algorithms for postoperative spine infection remain less clearly defined. A comprehensive understanding of the microbiologic patterns underlying spine infection is therefore essential to guide prevention strategies and optimize antimicrobial management.

## Credit author statement

AZ: Writing – original draft, Formal analysis.

WM: Investigation, Formal analysis, Data Curation, Writing – review & editing.

JT: Investigation.

SS: Investigation.

HS: Methodology, Data curation, Writing – review & editing.

WVV: Conceptualization, Writing – review & editing.

SK: Conceptualization, Writing – review & editing.

HL: Conceptualization, Methodology, Formal Analysis, Writing – review & editing.

## Ethical statement

All patient information was de-identified and patient consent was not required. All procedures were performed in compliance with relevant laws and institutional guidelines and have been approved by the appropriate institutional committee(s).

## Guardian/patient's consent

The following study is not a case report, nor does it have clinical images or private health information of patients. Therefore consent was not needed, however the study was conducted following the approval of our institution's IRB.

## Funding

This research did not receive any specific grant from funding agencies in the public, commercial, or not-for-profit sectors.

## Declaration of competing interest

The authors declare that they have no known competing financial interests or personal relationships that could have appeared to influence the work reported in this paper.
